# Nanoformulation of Polymyxin E Through Complex Coacervation: A Pharmacokinetic Analysis

**DOI:** 10.3390/pharmaceutics17010076

**Published:** 2025-01-08

**Authors:** Xiaobao Chen, Li Liu, Weidan Wang, Yuan Yuan, Wei Wang

**Affiliations:** 1 Scindy Pharmaceutical Co., Ltd., Suzhou Industrial Park, Suzhou 215125, China; liuli@scindypharm.com (L.L.);; 2 Center for Pharmacy, University of Bergen, 5020 Bergen, Norway; 3 Department of Chemistry, University of Bergen, 5007 Bergen, Norway

**Keywords:** antibiotics, nanoformulation, complex coacervation, PME, polymyxin E

## Abstract

**Objectives:** Polymyxin E (PME), a polymyxin antibiotic, serves as a final resort against antibiotic resistance. Nephrotoxicity is the primary concern when employing PME. To alleviate this issue, researchers have explored strategies including dosing adjustments and innovative formulations. This study employed complex coacervation to create PME nanoformulations, capitalizing on PME’s charge properties. The research question and hypothesis posed pertained to whether neutralization of PME’s positive charge during formulation would reduce its antibiotic efficacy and alter its tissue distribution and other pharmacokinetic parameters. Our objective was to evaluate the capability of complex coacervation to mitigate the adverse effects of PME while preserving its antibacterial potency and therapeutic effectiveness. **Methods:** Three negatively charged polyions: potassium sucrose octasulfate, polytamic acid, and sodium hyaluronate, were used for formulation. We performed characterization on the nanocomplex formed by the polyions and PME. The nanoformulations underwent several tests, including minimum inhibitory concentration, in vivo efficacy on an infected mouse model, pharmacokinetic assessments, tissue distribution, and toxicity. **Results:** the three polyions formed coacervation complexes with PME at varying charge ratios, yielding nanoparticles smaller than 30 nm with low polydispersity (PDI < 0.3). The results demonstrated that complex coacervation-mediated PME nanoformulations exhibited equivalent or superior antibacterial activity, increased maximum tolerant dose, and fewer adverse reactions in animal tests. **Conclusions:** Utilizing complex coacervation, PME nanoformulations were developed, demonstrating efficacy in the formulation process. Pharmacokinetic assessments revealed absorption and distribution profiles akin to those of standalone PME. The positive charge inherent in PME causing its toxicity was mitigated after complex coacervation.

## 1. Introduction

Antibiotic resistance represents a formidable challenge in our society. This phenomenon has been exacerbated by the misuse of antibiotics in human healthcare and agricultural practices, fostering the emerging of resistant strains [1]. Consequently, formerly manageable infections are increasingly defying conventional treatment approaches, thereby posing a profound and escalating threat to public health [2]. PME, a member of the polymyxin class of antibiotics, stands as a last-resort therapeutic option in the context of antibiotic resistance [3]. It is reserved for addressing infections caused by intractable Gram-negative bacterial strains that exhibit multidrug resistance, including resistance to a broad spectrum of antibiotics [4].

**Scheme 1:**
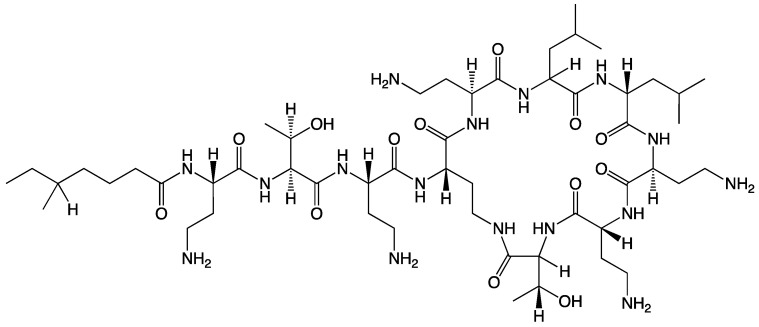
The chemical structure of PME.

PME possesses a distinctive chemical structure crucial to its antibacterial mechanism (Scheme 1) [5]. Its chemical makeup includes a cyclic heptapeptide with a tripeptide side chain that is acylated by a fatty acid at the amino terminus. Although closely related to polymyxin B, PME’s primary structural difference lies at position 6, where D-Phe (D-phenylalanine) is replaced with D-Leu (D-leucine). The structural elements encompass an intramolecular cyclic heptapeptide loop, connecting the side chain’s amino group on the diaminobutyric acid (Dab) residue at position 4 to the carboxyl group of the C-terminal L-Thr (L-threonine) residue at position 10. This forms the decapeptide sequence of PME, which includes the heptapeptide loop, the tripeptide side chain, and the fatty acid chain [6]. Moreover, PME exhibits characteristic features including cationic (L-α-γ-Dab) residues that render it polycationic at a neutral pH (7.4) and two hydrophobic regions—the N-terminal fatty acyl chain and the D-Leu6-L-Leu7 segment. These structural components contribute to PME’s amphipathic nature [7]. Moreover, 3D NMR analysis has unveiled how the PME molecule folds to create two distinct surfaces for its hydrophilic and hydrophobic domains, imparting structural amphipathicity essential for its antibacterial efficacy [8]. The mechanism of action primarily involves disrupting bacterial cell membranes, particularly in Gram-negative bacteria, by binding to the negatively charged lipopolysaccharide layer on their outer membrane. This disruption of cell membrane integrity is a key aspect of PME’s antibacterial mechanism, ultimately leading to bacterial cell death [9].

The antimicrobial action of PME hinges on its ability to disrupt the outer membrane of bacterial cells, based on the positively charged nature of the chemical structure [10]. Nevertheless, its clinical utilization is restricted due to the risk of adverse effects (i.e., nephrotoxicity) and the emergence of resistance mechanisms. To effectively address the complex and growing issue of antibiotic resistance, a cautious approach to PME delivery in clinical settings is imperative [11]. Simultaneously, there is a pressing need for extensive research aimed at discovering new formulations to reduce the side effects. This dual strategy underscores the importance of a comprehensive and interdisciplinary response to protect the effectiveness of PME. By advancing novel formulations, we can better confront the challenges posed by antibiotic-resistant bacteria. To mitigate nephrotoxicity, researchers have explored effective strategies such as adjusting dosing regimens [12] using liposomal formulations [13] or employing newer derivatives of polymyxins [14,15]. Alternative approaches for innovative formulation methods exist, yet they remain at a very early stage, distant from clinical implementation [16].

Complex coacervation, a phase separation phenomenon arising from the electrostatic interaction between oppositely charged polyions or molecules, has been widely employed in drug delivery for the encapsulation and preservation of diverse compounds [17]. However, its application in formulating PME to mitigate nephrotoxicity while preserving the drug’s antibacterial activity remains unexplored [18]. PME’s nephrotoxicity predominantly results from its cationic nature, leading to interactions with renal tubular cells, while maintaining its positive charge is essential for binding to bacteria. Striking a balance between its antimicrobial effectiveness and potential interaction with human cells is a critical aspect of PME’s formulation. The notion of neutralizing PME’s positive charge via complex coacervation was investigated in this study. We aimed to assess the potential of complex coacervation to reduce the side effects of PME while maintaining its antibacterial activity and therapeutic efficacy.

## 2. Materials and Methods

### 2.1. Chemicals and Reagents

**Scheme 2:**
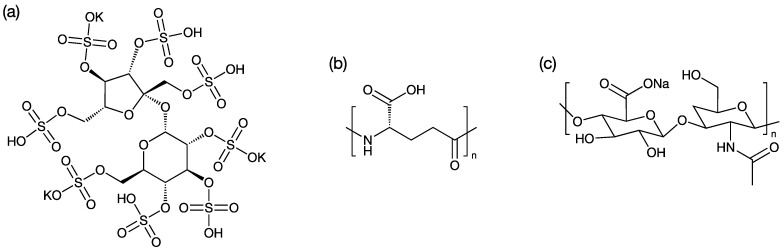
The chemical structures of (**a**) potassium sucrose octasulfate; (**b**) polyglutamic acid; (**c**) sodium hyaluronates.

PME was purchased from Hebei Shengxue Dacheng Tangshan Pharmaceutical Co., Ltd. Potassium sucrose octasulfate (SOP, Scheme 2a) was purchased from A.V.T. (Shanghai) Pharmaceutical Co., Ltd. (Shanghai, China). N-(carbonyl methoxypolyethylene glycol-2000)-1,2-distearoyl-sn-glycero-3-phosphoethanolamine (MPEG2000-DSPE) was purchased from Lipoid GmbH (Ludwigshafen, Germany). Polyglutamic acid (PGA, Scheme 2b) was purchased from Ricentik Co., Ltd. (Wuhan, China). Two sodium hyaluronates (HA, Scheme 2c) with average molecular weights of 1000k (HA1) and 24k (HA2) were purchased from Jiangsu Haihua Bio-Technology Co., Ltd. (Zhengjiang, China). All the chemicals were pharmaceutical grade.

### 2.2. Formulation of the PME–Polyion Coacervation Complex (PME Nanoformulation)

We dissolved PME and a polyion solution comprising HA, PGA, or SOP in water to form Solution 1, in which the concentration of PME was 3 mg/mL. We prepared Solution 2 by dissolving MPEG2000-DSPE in water. The two solutions were combined via a microfluidic device featuring a T-shaped channel, operating at a flow rate of 20 mL/min. The resulting mixture was subsequently filtered through a 0.22 µm sterilizing filter and stored in a sterile container for subsequent analysis. Seven nanoformulations were designed and synthesized using this method, and these formulations were named after the polyions used in the formulation and the charge ration between the polyions and the PME. The charge ratio and molar ratio for each nanoformulation are listed in Table 1. After preparation, the size and zeta potential of the nanoformulations were characterized using a Malvern NanoSizer according to the standard protocol provided by the supplier. The measurements were conducted following US pharmacopoeia specifications. All the measurements were conducted three times in parallel. The data are available in the Supporting Information (Table S1).

### 2.3. The Minimum Inhibitory Concentration (MIC) Test

In line with the 2021 edition of the United States Clinical and Laboratory Standards Institute (CLSI) guidelines (https://clsi.org/), we employed the broth microdilution method to determine the MIC of the compounds against clinical Gram-negative bacilli [19]. We tested 23 strains of *Escherichia coli*, including 12 carbapenem-sensitive and 11 drug-resistant strains, and 22 strains of *Klebsiella pneumoniae*, including 12 carbapenem-sensitive and 10 drug-resistant strains. Additionally, we assessed 20 strains of *Acinetobacter baumannii*, again including 10 carbapenem-sensitive and 10 drug-resistant strains, and 21 strains of *Pseudomonas aeruginosa*. Quality control strains used were *Escherichia coli* ATCC 25922 and *Pseudomonas aeruginosa* ATCC 27853 [20]. For the preparation of antimicrobial agents, we dissolved seven batches of the test drug in 3 mL of water and subsequently diluted it to a concentration of 500 mg/L. The control drug was prepared according to CLSI requirements. The antibacterial solutions were tested over a concentration range spanning from 0.015 mg/L to 32 mg/L. We utilized cation-adjusted Mueller–Hinton broth (CAMHB) from BD Company, with lot number 9324795.

Regarding bacterial inoculation, we selected fresh, pure bacterial colonies from Columbia blood agar using the direct colony suspension method, resulting in a turbidity of 0.5 McFarland units. This suspension was then appropriately diluted, yielding a final bacterial concentration of 10^5^ CFU/mL. Culturing conditions involved incubation in ambient air at 35 ± 2 °C for a duration of 16–20 h. Results were interpreted in accordance with the European EUCAST breakpoint criteria, where PME sensitivity was defined as ≤2 mg/L and resistance as ≥4 mg/L. Criteria for assessing other tested drugs were provided by the party commissioning the study. The MIC value represented the minimum drug concentration necessary to inhibit bacterial growth. Data analysis was performed using WHONET 5.6 software for statistical evaluation of the drug susceptibility test results.

### 2.4. Determination of Maximum Tolerated Dose (MTD)

CD-1 mice, male, 7–8 weeks old, weighing 25–30 g, were purchased from Shanghai Lingchang Laboratory Animal Co. The mice were grouped into n = 3, and different doses of PME were injected into the tail vein of mice via BID (q8h). HA2 prescription (HA2-2.6, HA2-2 and HA2-1.5) and SOP prescription (SOP-2.6, SOP-2 and SOP-1.5) were used for MTD examination. Mice were observed for survival and time to death was recorded. The dose that did not cause death in mice without serious adverse effects was defined as MTD.

### 2.5. In Vivo Efficacy of the Nanoformulation

CD-1 mice (SPF grade, female, 7 weeks old, 27–29 g) were provided by Zhejiang Weitong Lihua Laboratory Animal Technology Co., Ltd. A. Baumannii ATCC BAA-1605 was provided by Nantong Wuxi Apptec Medical Technology Co., Ltd. (Nanjing, China). Experimental animals were admitted to Wuxi AppTec BSL-2 animal facility and subjected to an adaptation period of at least 3 to 4 days. Immunosuppressed mice with neutrophil deficiency were prepared by intraperitoneal injection of cyclophosphamide (150 mg/kg at day -4; 100 mg/kg at day -1). On day 0, the infected bacterial solution was inoculated by intramuscular injection, and 0.1 mL of the bacterial solution was inoculated into the right thigh of each mouse. At 2 h and 24 h after infection, the PME formulations were administered by tail vein according to the experimental protocol. Animals in the blank group were euthanized 2 h after infection. The right thighs of those mice were collected and homogenized. The diluent was cultured overnight and CFUs were counted the next day. Animals in the negative control group and other treatment groups were euthanized 24 h after infection. The right thighs of those mice were also collected and homogenized. The diluent was cultured overnight and CFUs were counted the next day.

The dosing regimens for each experimental group are summarized as follows:

- Group 1: Blank control. No treatment administered.
- Group 2: Negative control. Dosage: 10 mL/kg; administration: twice daily (BID), every 12 h; no drug substance was added.
- Group 3: Excipient control. Dosage: equivalent to 32 mpk; administration: twice daily (BID), every 12 h; an excipient formulation was used.
- Group 4: Positive Control. Dosage: 8 mpk; administration: twice daily (BID), every 12 h; PME was administered at a dose of 8 mpk.
- Group 5–8: SOP-3. Dosages: 8 mpk, 16 mpk, 24 mpk, and 32 mpk; administration: twice daily (BID), every 12 h; different dosages of the SOP-3 formulation were administered.
- Groups 9–10: SOP-2. Dosages: 8 mpk and 24 mpk; administration: twice daily (BID), every 12 h; SOP-2 formulation at varying dosages was administered.
- Groups 11–12: HA2-1.5. Dosages: 8 mpk and 24 mpk; administration: twice daily (BID), every 12 h; HA2-1.5 formulation was administered at differing dosages.

The dosages were calculated based on the weight of the mice, and different dosage levels were achieved by adjusting the drug volume accordingly.

### 2.6. Pharmacokinetic Studies

SD rats (6–7 weeks old) were purchased from Shanghai Jihui Experimental Animal Feeding Co., Ltd. After the completion of administration, the time was recorded and blood samples were collected according to the set time point. Blood was collected from the jugular veins of the rats, added into 1.5 mL EDTA-K2 anticoagulant tube, thoroughly mixed, and placed on ice. Plasma was centrifuged within 30 min (4 °C, 5 min at 8000 rpm) and stored at −80 °C until determination. About 2 mL of blood was taken from the hearts of unadministered blank animals after carbon dioxide euthanasia and used as blank matrix plasma. To prepare the sample, 20 µL of the plasma was mixed with 50 µL of the internal standard and 200 µL of acetonitrile containing 1 M HCl. The mixture was vortexed and centrifuged, and the resulting supernatant was used for HPLC analysis. For HPLC measurement, a chromatographic column with dimensions l = 0.1 m, diameter = 4.6 mm was used, packed with end-capped C18 silica gel (Venusil MP C18), and maintained at 40 °C. The mobile phase consisted of 22 volumes of acetonitrile mixed with 78 volumes of a solution prepared by dissolving 4.46 g of anhydrous sodium sulfate in 900 mL of water, adjusting the pH to 2.4, and diluting to 1000 mL with water. The flow rate was 0.7 mL/min and detection was carried out at 215 nm using a spectrophotometer. The injection volume for analysis was 20 µL.

To assess PME distribution in tissues, SD rats, 7 weeks old, were purchased from Shanghai Jihui Experimental Animal Feeding Co., Ltd. After administration, routine examination included observing the effect of the drug on the animal’s daily behavior, including as behavioral activity, mental state, and water intake. Blood was collected from the heart after euthanasia, and the whole brain, heart, liver, spleen, lung, kidney, testis (including epididymis), ovary (including uterus), stomach wall, small intestine (jejunum), skin, skeletal muscle, and fat (abdomen) were collected immediately, along with partial plasma collection (at least 200 µL, centrifugation condition: at least 5000 rpm, 5 min). The residual blood and contents on the surface of each tissue were washed with cold normal saline. After drying, the tissue was labeled and stored at −70 °C for testing. The tissue sampling process was carried out at room temperature and samples were stored in an ultra-low temperature refrigerator as soon as possible after bagging. Blank tissue was collected from 6 other animals (3 male and 3 female) (the same type of tissue as above).

### 2.7. Toxicity of PME Nanoformulation

Male Balb/c mice, aged 6–7 weeks, were used for the toxicity test. The experimental design included three groups: blank control (only media), PME group (positive control, 10 mpk), and SOP group (10 mpk), each comprising 6 mice. Administered through the tail vein at a concentration of 1.5 mg/mL, the drug was given continuously for 7 days with a frequency of BID, q12h. Throughout the administration period, the mice underwent assessment of weight, food intake, and related changes. Urine was collected post-administration for plasma biochemistry and urine analysis.

## 3. Results

### 3.1. PME-Polyion Coacervation Complex

**Figure 1:**
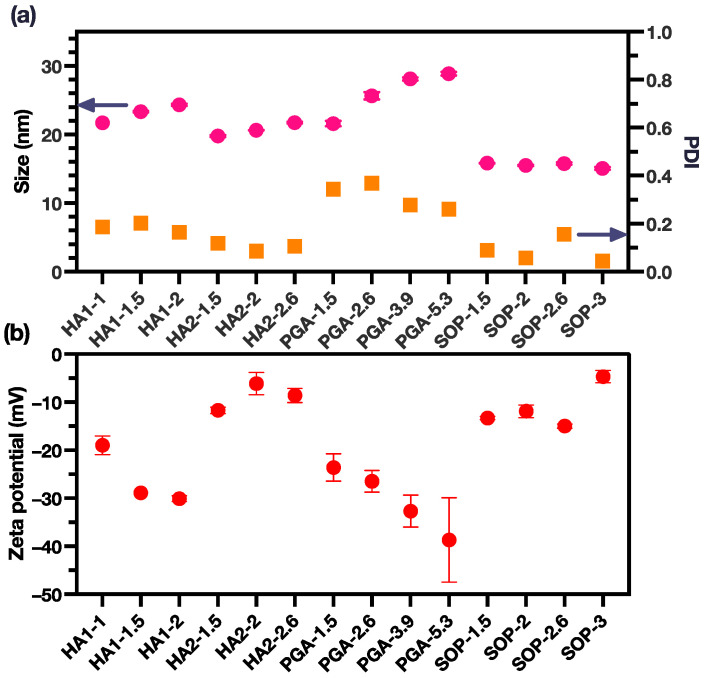
(**a**) The size and PDI of the PME nanoparticles (pink dots for size and yellow square for PDI); (**b**) The zeta potential of the PME nanoparticles (the data are reported as mean ± SD).

Three polyions, namely HA, PGA, and SOP, were chosen for the formation of coacervation-complex nanoformulations with PME. In this study, two distinct types of HA, characterized by varying average molecular weights, were utilized. As a result, our investigation encompassed the scrutiny of four sets of coacervation-complex nanoformulations. In each set, we systematically explored a minimum of three charge ratios for the polyions in relation to PME. All these polyions carried anionic charges and effectively formed coacervation complexes with the PME. The sizes of these coacervation complexes were observed to fall within the range of 15–30 nm (Figure 1a, Table S1). Furthermore, these complex sizes exhibited variability that was inherently contingent upon the specific charge ratio established between the polyions and the PME.

The sizes of the coacervation complexes displayed variations that were directly correlated with the charge ratio between the polyions and PME. For the HA1, HA2, and PGA groups, a discernible increase in complex size was evident with an escalating charge ratio. In the case of the SOP group, the size of the coacervation complex remained relatively constant, with only minor disparities observed among distinct groups. Notably, the SOP groups demonstrated the smallest particle size, approximately 15 nm, along with a low polydispersity index (PDI) below 0.2, signifying a monodisperse arrangement. In contrast, the PGA group exhibited the largest particle size, nearing 30 nm, along with the highest PDI values, which ranged from 0.2 to 0.4.

Within the HA groups, we highlight that HA1 possessed a significantly higher average molecular weight in comparison to HA2. Consequently, the coacervation complexes formed by HA1 were marginally larger than those formed by HA2. An increase in the charge ratio led to a more pronounced increase in size for the HA1 group compared with the HA2 group. Importantly, the PDI values for the HA1 and HA2 groups both consistently remained below the 0.2 threshold.

The morphologies of the nanoformulations were characterized using a transition electron microscope (TEM). The results are presented in Figure S1. All the nanoformulations were spherical in shape, and the size of the nanoparticles was consistent with the DLS measurements.

Zeta potential analyses revealed that all coacervation complexes exhibited a net negative charge. This observation underscored the effective neutralization of the positive charges originally present on the PME, with the complexes being enveloped by the negatively charged polyions (Figure 1b). The HA2 and SOP groups both exhibited the least negative zeta potential around −10 mV. Variations in the zeta potentials within these two groups remained relatively consistent across different charge ratios. In contrast, the PGA group showed the most pronounced surface charges, with zeta potentials ranging from −20 to −40 mV, and the surface charge becoming more negative as the charge ratio increased. It was also noted that the HA1 group displayed a more negative zeta potential in comparison to the HA2 group, probably attributable to the higher molecular weight associated with HA1.

### 3.2. Antimicrobial Activity of PME Nanoformulations

To delve deeper into the assessment of the antimicrobial efficacy of the coacervation-complex formulations, a selection of seven nanoformulations (namely PGA-2.6, HA2-2.6, HA2-2, HA2-1.5, SOP-2.6, SOP-2, and SOP-1.5) were designated for microbial inhibitory concentration (MIC) experiments, conducted in conjunction with a positive control (utilizing a PME solution). The MIC experiments were executed using a spectrum of bacterial strains, including Escherichia coli, Klebsiella pneumoniae, Acinetobacter baumannii, and Pseudomonas aeruginosa.

Table 2 presents the results of the MIC experiments for the seven nanoformulations and PME against two categories of *E. coli* isolates: carbapenem-sensitive (12 isolates) and carbapenem-resistant (11 isolates). For the carbapenem-sensitive and carbapenem-resistant *E. coli* isolates, the MIC range for all formulations was within ≤0.015 to 4 mg/L, indicating a wide range of susceptibility or resistance. For most formulations and both types of isolates, the MIC_50_ values were in the range from 0.03 mg/L to 0.125 mg/L, except for PME, which had higher MIC_50_ values. For all tested formulations, carbapenem-sensitive *E. coli* isolates showed no resistance (0%) and were completely susceptible (100%). Carbapenem-resistant *E. coli* isolates also showed no resistance (0%) to most formulations but exhibited some resistance (9.1%) to PME. The majority (90.9%) of the carbapenem-resistant isolates were susceptible to PME. PME, on the other hand, exhibited some resistance to the carbapenem-resistant isolates (9.1%) but still retained good efficacy (90.9% susceptibility) against this challenging group of bacteria. All the developed formulations demonstrated better efficacy against both carbapenem-sensitive and carbapenem-resistant E. coli isolates compared with PME alone, as indicated by their low MIC_50_ values and the absence of resistance.

Table 3 shows the results of the MIC experiments for the same formulations against two categories of *K. pneumoniae*: carbapenem-sensitive (12 isolates) and carbapenem-resistant (10 isolates). The MIC values for all formulations ranged from 0.03 to 0.5 mg/L, indicating a wide range of susceptibility/resistance. For all formulations, both carbapenem-sensitive and carbapenem-resistant isolates showed 0% resistance and 100% susceptibility. This suggests that, based on MIC values alone, none of the tested isolates were resistant to the formulations. The MIC results indicated that all the tested isolates, whether carbapenem-sensitive or carbapenem-resistant, exhibited a high degree of susceptibility to the tested formulations. This suggests that the formulations had potent antimicrobial activity against both types of *K. pneumoniae*. The MIC_50_ values for carbapenem-sensitive isolates were consistent across all formulations, indicating a uniform response to the different antimicrobial agents. For carbapenem-resistant isolates, the MIC_50_ remained constant at 0.06 mg/L, suggesting consistent activity against the majority of these isolates. The absence of any resistant isolates in the experiment was a positive outcome, suggesting that these formulations may be effective in treating infections caused by these *K. pneumoniae* strains.

Table 4 presents the results of the MIC experiments for the formulations against two groups of *A. baumannii* isolates: carbapenem-sensitive (10 isolates) and carbapenem-resistant strains (10 isolates). For all formulations tested, the MIC range was broad, ranging from 0.06 to 1 mg/L, indicating that the susceptibility of A. baumannii isolates to these formulations varied widely. MIC_50_ values varied between formulations but generally remained low, indicating that the formulations were effective in inhibiting bacterial growth for most isolates. The data indicated that none of the isolates showed resistance to any of the formulations, as indicated by a 0% resistance rate. Conversely, all isolates were susceptible to all formulations, with a 100% susceptibility rate. This suggests that the formulations had a strong inhibitory effect on both the carbapenem-sensitive and carbapenem-resistant *A. baumannii* strains. Comparing the different formulations, it appears that they had similar MIC_50_ values, suggesting that they exhibited similar effectiveness against the tested isolates. However, there were some minor variations, such as PME having slightly higher MIC_50_ values compared with the other formulations. In the overall analysis, the formulations maintained their effectiveness across both carbapenem-sensitive and carbapenem-resistant strains, with 100% susceptibility and no observed resistance.

Table 5 shows the results of the MIC experiments against carbapenem-sensitive *P. aeruginosa* (10 isolates) and carbapenem-resistant *P. aeruginosa* (11 isolates). Across all the isolates, the MIC range for most formulations was 0.03 to 4 mg/L. The MIC_50_ was typically 0.5 mg/L. The rate of resistance was 0% for most formulations, indicating a lack of resistance among the isolates. HA2-1.5 and SOP-1.5 showed a higher percentage of resistance (4.8%) compared with the other formulations. The susceptible percentage was generally high, ranging from 95.2% to 100%. The formulations generally exhibited good antimicrobial activity against both the carbapenem-sensitive and carbapenem-resistant *P. aeruginosa* isolates, as indicated by low MIC values. There was no significant difference in susceptibility between the two categories of *P. aeruginosa* for most formulations. Formulations HA2-1.5 and SOP-1.5 showed slightly higher levels of resistance in their carbapenem-resistant isolates, but the majority of isolates remained susceptible. Overall, the results suggest that these nanoformulations have potential as antimicrobial agents against *P. aeruginosa*, particularly for carbapenem-sensitive strains.

### 3.3. MTD

**Figure 2:**
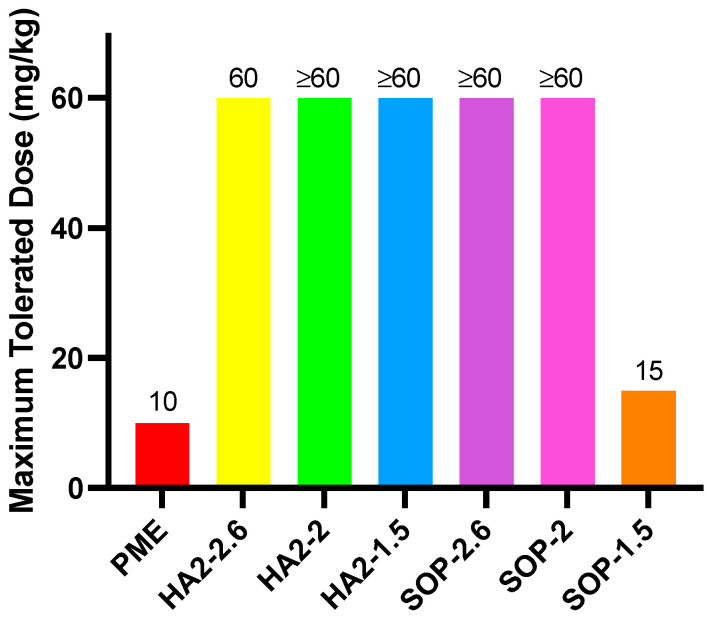
MTD results for PME, HA2, and SOP nanoformulations.

Figure 2 illustrates that the MTD for free PME was 10 mpk, with one mouse succumbing within 30 min after the dose was increased to 12 mpk. In the SOP-1.5 prescription group, the MTD was approximately 15 mpk, but when the dose was raised to 20 mpk, one mouse died within 10 min. SOP-2, HA2-2, and HA2-1.5 prescriptions were tolerated up to 60 mpk, with some clinical effects observed. At this dose, HA2-2 and HA2-1.5 resulted in reduced activity, shortness of breath, and eventual prostration; in addition, mice presented with slowing of breath following an initial period of shortness. Conversely, when SOP-2.6 was administered at 60 mpk, no significant clinical abnormalities were observed. These findings indicated that for the SOP prescription series, increasing the SOP dose markedly decreased toxicity, while for the HA series, toxicity showed no consistent relationship with dosage level.

### 3.4. In Vivo Efficacy of PME Nanoformulations

**Figure 3:**
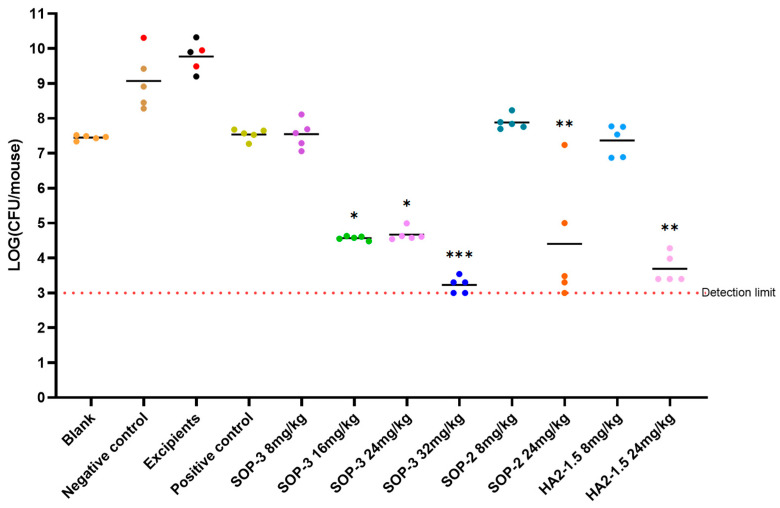
Antibacterial efficacy of three nanoformulations on mouse model inoculated with A.baumannii ATCC BAA-1605 at 2.7 × 10^6^ CFU/mouse through i.m. (n = 5 in each group). Note: *** compared with negative control group, *p* < 0.001, with highly significant difference; ** compared with negative control group, *p* < 0.01, with highly significant difference; * compared with the negative control group, *p* < 0.05 with significant difference, via one-way ANOVA. The bar for each group presents the mean value for that group.

In order to further evaluate the nanoformulations for clinical application, carbapenem resistant *Acinetobacter baumannii* (ATCC BAA-1605) was used to construct a stable mouse thigh-muscle infection model and evaluate the antibacterial activity of the PME nanoformulations in vivo. We conducted a pharmacological test to assess the efficacy of various drug formulations against carbapenem-resistant *A. baumannii* infection in mouse muscle (Figure 3). The effectiveness of three nanoformulations (SOP-3, SOP-2, HA2-1.5) was evaluated together with a positive control, negative control, and excipient control. For the three nanoformulations, we also studied the antibacterial effects of dose dependency.

The negative control and excipient control groups saw a notable rise in bacterial levels in the mice 24 h after infection. This led to severe alopecia, reduced mobility, and in some cases, fatalities. Among these, the excipient control group had the highest bacterial load at 8.53 × 10^9^ CFU/mouse, indicating a significant bacterial infection. The positive control (8 mpk) did not increase bacterial levels but did cause severe alopecia.

At lower dosages, the antibacterial capabilities of these three nanoformulations matched those of the positive control compound, PME. The SOP-3 nanoformulation at 8 mpk showed a bacterial load of 4.95 × 10^7^ CFU/mouse, indicating substantial antibacterial activity without observable symptoms. As we increased the dosage (16 mpk, 24 mpk, and 32 mpk), the bacterial load continued to decrease significantly, suggesting a dose-dependent response. In essence, as we increased the dosage of the examined nanoformulations, we observed a corresponding improvement in their antibacterial effectiveness. The SOP-3 and HA2-1.5 nanoformulations both demonstrated strong antibacterial effects at 16 mpk and above, with no signs of toxicity.

### 3.5. Pharmacokinetic Study

**Figure 4:**
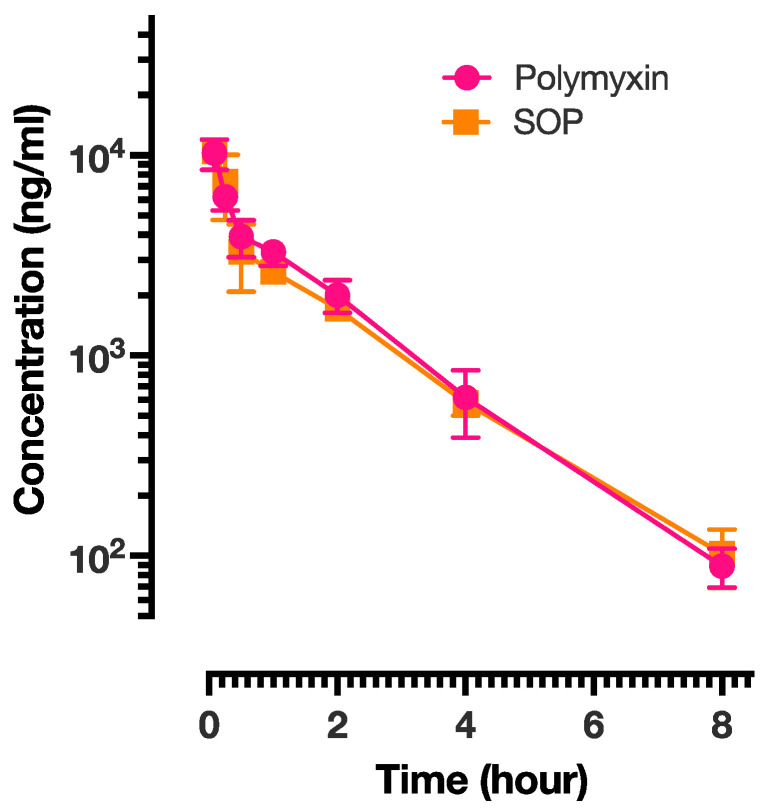
The plasma concentration of PME presented as a function of time for PME and SOP-3 nanoformulations. The data points are presented as mean ± SD.

Pharmacokinetic studies were performed on the PME and SOP-3 nanoformulations. The results are presented in Figure 4 and the analysis is shown in Table 6. PME exhibited the shortest half-life (T_1/2_), suggesting relatively rapid elimination from the body, while SOP had a slightly longer half-life, indicating a slower elimination rate. All three formulations achieved their maximum concentration within a short time (0.08 h or approximately 5 min) after administration. Mean residence time (MRT) represents the average duration a drug molecule remains in the body. The PME and SOP nanoformulations showed similar MRT values, implying comparable residence times within the body. SOP demonstrated a higher volume of distribution at steady state (Vss), suggesting a larger distribution volume in comparison to PME. This indicates a potentially broader tissue distribution. There was no significant difference in the exposure of the two formulations in rats. SOP also exhibited higher C_max_ and AUC values, indicating a higher peak concentration and greater overall exposure. This could be attributed to a slightly lower clearance rate compared with the other two formulations. All three formulations followed a two-compartment model. The intersection points between the distribution phase and elimination phase for SOP and PME occurred at 0.5 h. Based on the pharmacokinetic results, it appeared that both formulations were predominantly distributed in body fluids, with limited tissue distribution. To verify this conclusion, tissue distribution of the PME and SOP nanoformulations was further investigated.

### 3.6. Tissue Distribution in PME Group and SOP Nanoformulation

**Figure 5:**
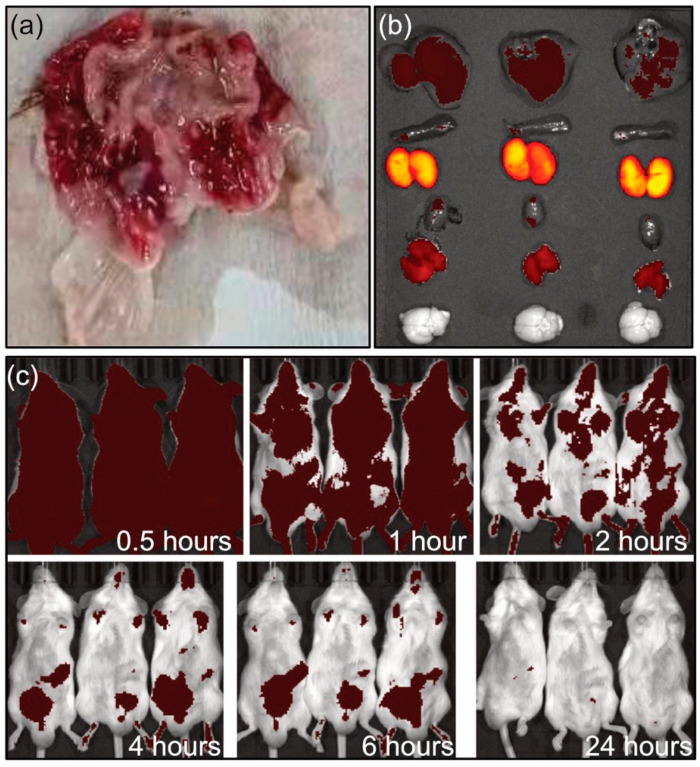
(**a**) Gastric bleeding in male group after 2 h intravenous administration of PME. (**b**) Imaging of organs for SOP group at 2 h (from top to bottom: lever, spleen, kidney heart, muscle, brain). (**c**) In vivo imaging at different times.

The tissue distribution of PME and SOP nanoformulations were studied. PME and SOP formulations were administrated by single intravenous injection. After the administration, all subjects showed decreased activity, increased water intake, and slight congestion of ears and limbs. At the end of the experiment, the anatomy of animals in the PME male subject group showed gastric mucosal bleeding at 0.5 h and 2 h (Figure 5a). The gastric mucosal bleeding in the male group at 0.5 h mainly occurred at the junction between the stomach body and the gastric pylorus, and the bleeding range was small. In the male group at 2 h, gastric mucosal bleeding occurred in most areas except the pylorus, and the range of bleeding was large. Gastric mucosal bleeding was not observed in the SOP group, indicating lower adverse reaction.

**Figure 6:**
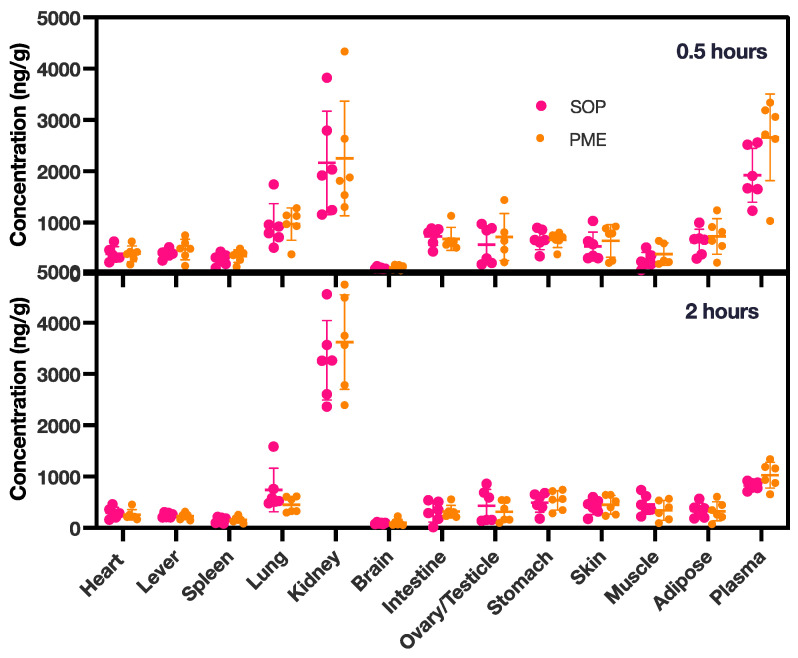
PME concentrations in various tissues at 0.5 and 2 h for both the PME and the SOP group. The study involved six animals, with an equal distribution of three females and three males. PME concentrations in plasma were measured in ng/mL. The data points are presented as mean ± SD.

In the tissue distribution experiment (Figure 5b,c and Figure 6), in both groups, the PME and SOP nanoformulations were mainly distributed in the kidneys after single intravenous administration, followed by the lungs. The concentration of PME was higher in the lungs at 0.5 h, due to the intravenous injection; however, it decreased quickly after 2 h. Other tissues except the brain also had small amounts distributed. With the extension of time, the kidney exposure increased slightly, and the exposure of the other organs decreased. In addition, compared with PME, the SOP nanoformulation had slightly lower exposure to the kidneys and slightly higher exposure to the other organs at 0.5 h (Figure 6), and increased exposure to the kidneys and decreased exposure to other organs at 2 h. However, the *p* value of the adipose tissue was less than 0.05 at 2 h. There was no significant difference in other tissues (*p* < 0.05). With the extension of time, the exposure of the PME and SOP nanoformulations to the kidneys increased to a certain extent, while exposure to the other tissues decreased, suggesting that the drug may have been excreted by kidney metabolism.

In vivo imaging and tissue distribution following single intravenous administration in ICR mice were investigated (Figure 5b,c). The results showed that the PME nanoformulation (SOP) labeled with Alexa Fluor™ 647 was metabolized rapidly in the body after intravenous injection. At 0.5 h, the drug was mainly distributed in the kidney tissues (Figure 5b), with a small amount in other tissues (such as liver and muscle). At 4 and 6 h, the drug was mainly concentrated in the abdominal bladder area, and it was completely excreted at 24 h (Figure 4 and Figure 5c). PME is metabolized by the kidneys and excreted in urine. The results of this study are basically consistent with the literature data [21,22]. The metabolic rate and pathway of the PME and SOP nanoformulations were similar, but with a lower adverse reaction for the SOP nanoformulation.

### 3.7. Toxicity of the SOP Nanoformulation

**Figure 7:**
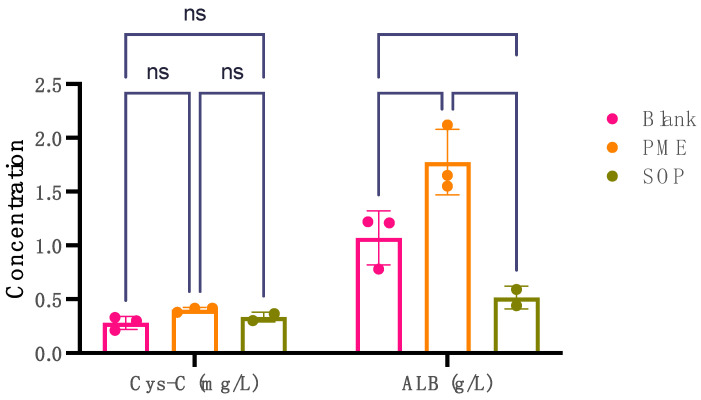
The levels of Cys-C in mg/L and ALB in g/L in urine for three groups: blank group, PME group, and SOP group. Note: **** compared with PME group, *p* < 0.0001; ** compared with blank group, *p* < 0.001; * compared with blank group, *p* < 0.01 with significant difference, two-way ANOVA. The data points are presented as mean ± SD.

Cystatin C (Cys-C) and albumin (ALB) levels serve as key markers in assessing nephrotoxicity. Elevated urinary levels of Cys-C and albumin are indicative of potential kidney damage or nephrotoxic effects. The comparison between the PME (10 mpk) group and the blank group revealed statistically significant findings in terms of Cys-C and ALB (Figure 7). Notably, the PME (10 mpk) group exhibited a significant increase in urinary Cys-C levels compared with the blank group, indicating potential kidney impairment such as damage to the proximal renal tbules. Moreover, a substantial rise in urinary ALB levels was observed in the PME group (*p* = 0.0025), suggesting possible kidney damage, as increased albumin excretion in urine can indicate renal dysfunction. Conversely, the SOP (10 mpk) group demonstrated no significant difference in urinary Cys-C levels compared with the blank group (*p* = 0.0227). Interestingly, a reduction in urinary ALB levels was noted (*p* < 0.0001), potentially signifying mitigation of kidney injury.

## 4. Conclusions

We employed complex coacervation to formulate PME with the aim of mitigating nephrotoxicity. We combined PME with three negatively charged polyions, resulting in the formation of nanoparticles ranging from 15 to 30 nm in size, displaying a narrow size distribution. These nanoparticles carried a negative charge, indicating PME embedded in the nanoparticles. Seven nanoformulations were subjected to MIC testing across a spectrum of bacterial strains, including Escherichia coli, Klebsiella pneumoniae, Acinetobacter baumannii, and Pseudomonas aeruginosa. The MIC results demonstrated that these nanoformulations possessed equal or superior antibacterial efficacy. MIC values ranged from 0.015 to >32 mg/L, reflecting a broad spectrum of susceptibility and resistance. In vivo testing on infected mouse models revealed a dose-dependent response to the SOP nanoformulation. The MTDs of both the HA2 and SOP nanoformulations in mice were differentially elevated compared with the PME, up to more than 6-fold. PK analysis indicated similar tissue distribution and excretion profiles among the PME and SOP nanoformulations. While gastric mucosal bleeding was observed with PME, it was absent in the case of the SOP nanoformulation. In vivo imaging demonstrated that PME predominantly accumulated in kidney tissues, with minimal presence in other tissues. At 4 and 6 h, the drug primarily localized in the abdominal bladder area and was fully excreted within 24 h. In summary, our findings reveal that complex coacervation-mediated PME nanoformulations exhibit equivalent or superior antibacterial activity, reduce renal exposure, and result in fewer adverse reactions.

## Figures and Tables

**Table 1:** The polyion–PME charge ratio and PME–polyion–MEPG2000-DSPE molar ratio.

	Charge Ratio of Polyion/PME	Molar Ratio of PME/Polyion/MPEG200-DSPE
HA1-1	1:1	1:0.002:5
HA1-1.5	1.5:1	1:0.003:5
HA1-2	2:1	1:0.004:5
HA2-1.5	1.5:1	1:0.12:6.73
HA2-2	2:1	1:0.17:6.98
HA2-2.6	2.6:1	1:0.22:7.28
PGA-1.5	1.5:1	1:0.12:5.23
PGA-2.6	2.6:1	1:0.22:7.28
PGA-3.9	3.9:1	1:0.33:7.93
PGA-5.3	5.3:1	1:0.43:8.58
SOP-1.5	1.5:1	1:0.93:5.35
SOP-2	2:1	1:1.25:6.24
SOP-2.6	2.6:1	1:1.63:7.28
SOP-3	3:1	1:1.88:6.00

**Table 2:** MIC range (mg/L), MIC50, resistance, and susceptibility of the formulations against 23 strains of *Escherichia coli*.

Organism (No. of Isolates)	Formulation	MIC Range	MIC_50_	Resistant (%)	Susceptible (%)
Carbapenem-sensitive *E. coli* (12)	PGA-2.6	≤0.015–0.06	0.03	0	100
	HA2-2.6	0.03–0.06	0.03	0	100
	HA2-2	≤0.015–0.06	0.03	0	100
	HA2-1.5	0.03–0.06	0.03	0	100
	SOP-2.6	0.03–0.06	0.06	0	100
	SOP-2	0.03–0.06	0.06	0	100
	SOP-1.5	0.03–0.125	0.06	0	100
	PME	0.125–0.5	0.125	0	100
Carbapenem-resistant *E. coli* (11)	PGA-2.6	0.03–2	0.06	0	100
	HA2-2.6	0.03–1	0.06	0	100
	HA2-2	0.03–1	0.06	0	100
	HA2-1.5	0.03–2	0.06	0	100
	SOP-2.6	0.06–1	0.06	0	100
	SOP-2	0.06–1	0.125	0	100
	SOP-1.5	0.06–2	0.06	0	100
	PME	0.125–4	0.25	9.1	90.9
Total (23)	PGA-2.6	≤0.015–2	0.06	0	100
	HA2-2.6	0.03–1	0.03	0	100
	HA2-2	≤0.015–1	0.06	0	100
	HA2-1.5	0.03–2	0.06	0	100
	SOP-2.6	0.03–1	0.06	0	100
	SOP-2	0.03–1	0.06	0	100
	SOP-1.5	0.03–2	0.06	0	100
	PME	0.125–4	0.25	4.3	95.7

**Table 3:** MIC range (mg/L), MIC50, resistance, and susceptibility of the formulations against 22 strains of Klebsiella pneumoniae.

Organism (No. of Isolates)	Formulation	MIC Range	MIC_50_	Resistant (%)	Susceptible (%)
carbapenem-sensitive *K. pneumoniae* (12)	PGA-2.6	0.06–0.25	0.06	0	100
	HA2-2.6	0.06–0.125	0.06	0	100
	HA2-2	0.06–0.125	0.06	0	100
	HA2-1.5	0.06–0.125	0.06	0	100
	SOP-2.6	0.06–0.125	0.125	0	100
	SOP-2	0.06–0.125	0.125	0	100
	SOP-1.5	>0.06–0.125	0.125	0	100
	PME	0.25–0.5	0.5	0	100
carbapenem-resistant *K. pneumoniae* (10)	PGA-2.6	0.03–0.125	0.06	0	100
	HA2-2.6	0.03–0.06	0.06	0	100
	HA2-2	0.03–0.125	0.06	0	100
	HA2-1.5	0.06–0.125	0.06	0	100
	SOP-2.6	0.06–0.125	0.06	0	100
	SOP-2	0.06–0.125	0.125	0	100
	SOP-1.5	0.06–0.5	0.125	0	100
	PME	0.125–0.5	0.5	0	100
Total (22)	PGA-2.6	0.03–0.25	0.06	0	100
	HA2-2.6	0.03–0.125	0.06	0	100
	HA2-2	0.03–0.125	0.06	0	100
	HA2-1.5	0.06–0.125	0.06	0	100
	SOP-2.6	0.06–0.125	0.06	0	100
	SOP-2	0.06–0.125	0.125	0	100
	SOP-1.5	0.06–0.5	0.125	0	100
	PME	0.125–0.5	0.5	0	100

**Table 4:** MIC range (mg/L), MIC50, resistance, and susceptibility of the formulations against 20 strains of Acinetobacter baumannii.

Organism (No. of Isolates)	Formulation	MIC range	MIC_50_	Resistant (%)	Susceptible (%)
Carbapenem-sensitive *A. baumannii* (10)	PGA-2.6	0.06–0.125	0.125	0	100
	HA2-2.6	0.06–0.125	0.125	0	100
	HA2-2	0.06–0.125	0.125	0	100
	HA2-1.5	0.06–0.25	0.125	0	100
	SOP-2.6	0.06–0.25	0.125	0	100
	SOP-2	0.125–0.5	0.25	0	100
	SOP-1.5	0.125–0.25	0.125	0	100
	PME	>0.25–0.5	0.5	0	100
Carbapenem-resistant *A. baumannii* (10)	PGA-2.6	0.125–0.5	0.25	0	100
	HA2-2.6	0.125–0.5	0.25	0	100
	HA2-2	0.125–0.5	0.25	0	100
	HA2-1.5	0.125–0.5	0.25	0	100
	SOP-2.6	0.25–0.5	0.25	0	100
	SOP-2	0.25–0.5	0.5	0	100
	SOP-1.5	0.25–0.5	0.25	0	100
	PME	0.5–1	0.5	0	100
Total (20)	PGA-2.6	0.06–0.5	0.125	0	100
	HA2-2.6	0.06–0.5	0.125	0	100
	HA2-2	0.06–0.5	0.125	0	100
	HA2-1.5	0.06–0.5	0.125	0	100
	SOP-2.6	0.06–0.5	0.25	0	100
	SOP-2	0.125–0.5	0.25	0	100
	SOP-1.5	0.125–0.5	0.25	0	100
	PME	0.5–1	0.5	0	100

**Table 5:** MIC range (mg/L), MIC_50_, resistance, and susceptibility of the formulations against 21 strains of Pseudomonas aeruginosa.

Organism (No. of Isolates)	Formulation	MIC Range	MIC_50_	Resistant (%)	Susceptible (%)
Carbapenem-sensitive *P. aeruginosa* (10)	PGA-2.6	0.03–1	0.25	0	100
	HA2-2.6	0.03–4	0.25	10	90
	HA2-2	0.06–1	0.25	0	100
	HA2-1.5	0.06–1	0.25	0	100
	SOP-2.6	0.06–2	0.5	0	100
	SOP-2	0.125–2	0.5	0	100
	SOP-1.5	0.06–2	0.25	0	100
	PME	0.125–2	1	0	100
Carbapenem-resistant *P. aeruginosa* (11)	PGA-2.6	0.125–2	1	0	100
	HA2-2.6	0.25–2	0.5	0	100
	HA2-2	0.125–2	1	0	100
	HA2-1.5	0.125–4	1	9.1	90.9
	SOP-2.6	0.25–2	1	0	100
	SOP-2	0.25–2	1	0	100
	SOP-1.5	0.25–4	1	9.1	90.9
	PME	1–2	2	0	100
Total (21)	PGA-2.6	0.03–2	0.5	0	100
	HA2-2.6	0.03–4	0.5	4.8	95.2
	HA2-2	0.06–2	0.5	0	100
	HA2-1.5	0.06–4	0.5	4.8	95.2
	SOP-2.6	0.06–2	0.5	0	100
	SOP-2	0.125–2	1	0	100
	SOP-1.5	0.06–4	0.5	4.8	95.2
	PME	0.125–2	2	0	100

**Table 6:** The pharmacokinetic parameters for PME and SOP nanoformulations. The data are presented as mean ± SD.

	T_1/2_ (h)	T_max_ (h)	C_max_ (μg/mL)	AUC (h•g/mL)	AUC_INF_ (h•μg/mL)	Cl (mL/min/kg)	MRT_INF_ (h)	Vss (mL/kg)
**PME**	1.33 ± 0.05	0.08	10.26 ± 1.78	12.12 ± 2.08	12.29 ± 2.12	4.16 ± 0.78	1.63 ± 0.15	403.50 ± 54.46
**SOP**	1.48 ± 0.19	0.08	10.50 ± 0.81	11.13 ± 0.92	11.35 ± 0.90	4.42 ± 0.34	1.69 ± 0.20	448.24 ± 68.00

## Data Availability

Data are unavailable due to privacy.

## Supplementary Materials

The following supporting information can be downloaded at https://www.mdpi.com/article/10.3390/pharmaceutics17010076/s1, Table S1: The size distribution, PDI, and zeta potential of the nanoformulations. Figure S1: The transmission electron microscope (TEM) images for (a) HA2-2.6; (b) PGA-2.6; (c) SOP-2.6. The observations were conducted using a field emission TEM. The microscope, model FEI F20, operated at an accelerating voltage of 200 kilovolts (kV) with a high resolution of 0.14 nm along with a point resolution of 0.24 nm. Figure S2: The size distribution of all the nanoformulations.
