# Supplementary material for: Nanoformulation of Polymyxin E Through Complex Coacervation: A Pharmacokinetic Analysis
Source: Pharmaceutics. 2025 Jan 8;17(1):76. doi: 10.3390/pharmaceutics17010076 (PMC11769286; doi:10.3390/pharmaceutics17010076)
Supplement: Supplementary file 1 [file pharmaceutics-17-00076-s001.zip › pharmaceutics-3325241-supplementary.pdf]

Supporting information for

Nanoformulation of Polymyxin E Through Complex Coacervation: A Pharmacokinetic Analysis

Xiaobao Chen [1,*], Li Liu [1], Weidan Wang [1], Yuan Yuan [1] and Wei Wang [2,3,*]

[1] Scindy Pharmaceutical Co., Ltd., Suzhou Industrial Park, Suzhou 215125, China; liuli@scindypharm.com (L.L.)
[2] Center for Pharmacy, University of Bergen, 5020 Bergen, Norway
[3] Department of Chemistry, University of Bergen, 5007 Bergen, Norway
* Correspondence: paulchen@scindypharm.com (X.C.); wei.wang@uib.no (W.W.)

Table S1. The size distribution, PDI and Zeta potential of the nanoformulations.

	Size (nm)	PDI	Zeta Potential (mV)
HA1-1	21.70±0.1365	0.186±0.003	-19.0±1.93
HA1-1.5	23.35±0.1168	0.202±0.009	-41.5±7.53
HA1-2	24.34±0.1652	0.164±0.014	-28.9±0872
HA2-1.5	19.80±0.1457	0.118±0.015	-11.7±0.656
HA2-2	20.64±0.02000	0.085±0.001	-6.15±2.34
HA2-2.6	21.74±0.1258	0.106±0.007	-8.62±1.49
PGA-1.5	21.61±0.3905	0.344±0.035	-23.6±2.82
PGA-2.6	25.66±0.5333	0.369±0.060	-26.5±2.25
PGA-3.9	28.12±0.2117	0.278±0.007	-32.7±3.33
PGA-5.3	28.89±0.2722	0.260±0.008	-38.7±8.79
SOP-1.5	15.84±0.03786	0.089±0.020	-13.3±0.361
SOP-2	15.50±0.08145	0.057±0.005	-11.9±1.31
SOP-2.6	15.77±0.1553	0.155±0.029	-15.0±0.451
SOP-3	15.07±0.1931	0.044±0.015	-4.68±1.29

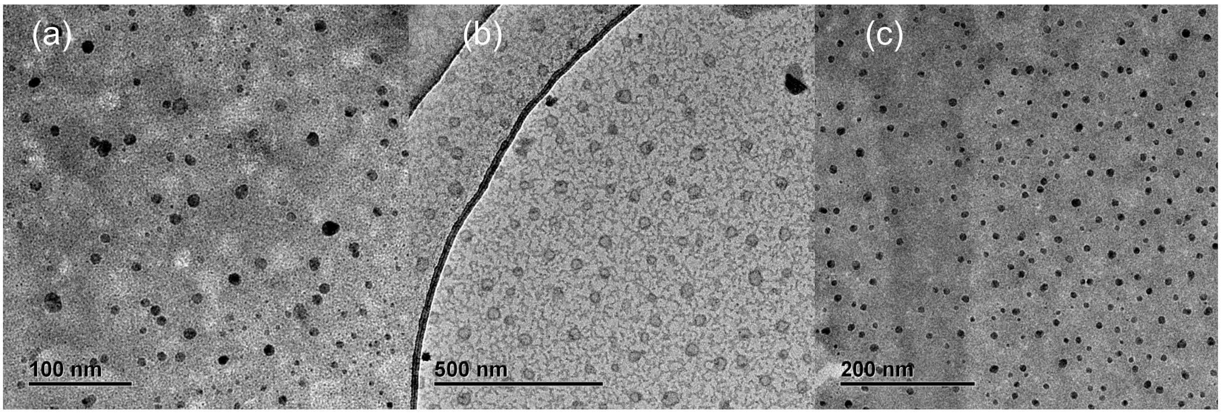

Figure S1. The transmission electron microscope (TEM) images for (a) HA2-2.6; (b) PGA-2.6; (c) SOP-2.6. The observation was conducted using a field emission TEM. The microscope, model FEI

F20, operates at an accelerating voltage of 200 kilovolts (kV) and boasts a high resolution of 0.14 nm along with a point resolution of 0.24 nm.

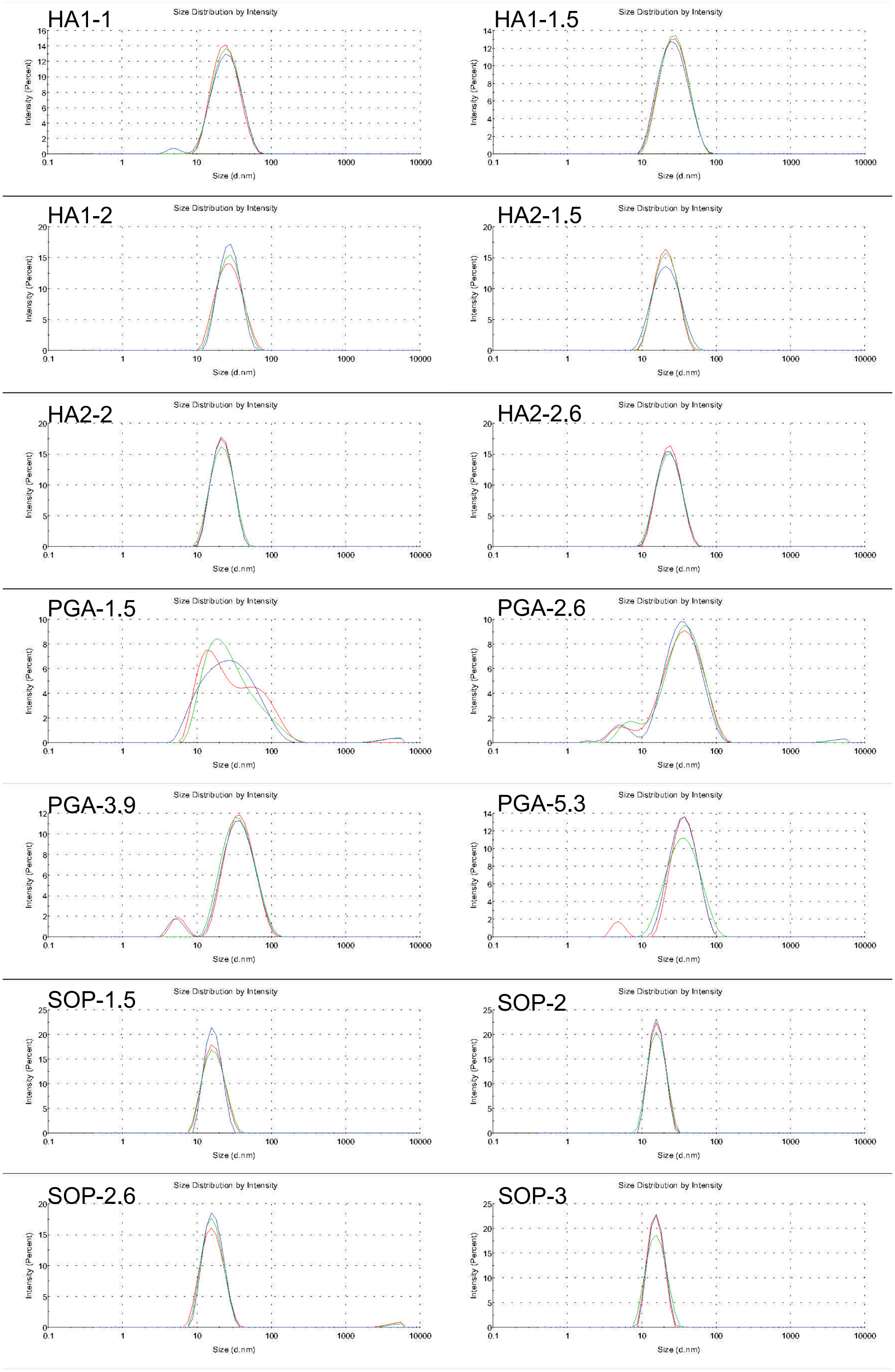

Figure S2. The size distribution of all nanoformulations.
